# Comparison of Commercially Available Arch Wires with Normal Dental Arch in a Group of Iranian Population

**Published:** 2015-06

**Authors:** Zohreh Hedayati, Farnaz Fakhri, Vahid Moshkel Gosha

**Affiliations:** 1 Orthodontic Research Center, Dept. of Orthodontics, School of Dentistry, Shiraz University of Medical Sciences, Shiraz, Iran;; 2 Postgraduate Student, Orthodontic Research Center, School of Dentistry, Shiraz University of Medical Sciences, Shiraz, Iran;

**Keywords:** Arch Wire, Dental Arch Form, Arch Size

## Abstract

**Statement of the Problem:**

The stability of orthodontic treatment depends on preserving the patient’s pretreatment arch form and arch size during and after treatment.

**Purpose:**

This investigation was aimed to study the size and shape of Iranian mandibular dental arch and evaluate the correlation of their average dental arch with commercially available preformed rectangular nickel-titanium arch wires.

**Materials and Method:**

In this study, 148 subjects were selected among students of Shiraz University of Medical Sciences. The inclusion criteria were having Angle class I in molar and canine relationships, and normal growth pattern. Intercanine and intermolar widths were measured after scanning their mandibular dental casts. Three main arch form templates; square, ovoid and tapered (Orthoform ^TM^; 3M, Unitek, CA, USA) and 12 commercially available preformed mandibular nickel-titanium arch wires were scanned. Intercanine and intermolar widths of arch wires were compared with dental arch widths of the study samples. Arch width, arch form and the most appropriate arch wire were determined for each cast. Student’s t-test was used to compare arch widths and arch depths of male and female subjects. Coefficient of variance was used to determine the variability of indices in the study samples.

**Results:**

Most preformed arch wires were wider than the average width of the normal Iranian dental arch. The most frequent arch form in Iranian population was tapered. Inter molar width was the only statistically significant variable between males and females.

**Conclusion:**

Variation in available preformed arch wires does not entirely cover the range of diversity of the normal dental arch of our population. Narrow arch wires with a tapered shape are better consistent with the Iranian lower arch.

## Introduction

The most important part of orthodontic treatment is aligning the teeth on the patient’s dental arches. Each patient has a special arch form and arch size. Stability of orthodontic treatment depends on preserving the patient’s pretreatment arch form and arch size during and at the end of treatment.[1-3]

Arch width and shape are important characteristics of the dental arch. Although different classifications of arch form have been suggested, three main arch forms (ovoid, tapered, square) are commonly used by the clinicians.[4] Arch perimeter, arch width and arch depth are used for arch size measurements. Inter-canine and inter-molar widths are accurate indices for showing muscle equilibrium.[5] Longitudinal studies have shown high probability of relapse after increasing arch width especially in the mandibular canine region.[6]

When the Edgewise technique was first introduced in the 1920s, bending the arch wires in order to match the dental arch was an important part of orthodontic treatment and dental casts were used in order to form arch wires.[7] Today, dental casts are replaced by 3-D digital models to produce prefabricated arch wires.[1, 8] Since the introduction of nickel-titanium wires, preformed types of these wires have been widely used, particularly in the initial phases of orthodontic treatment.[9] Invention of self-ligating and straight wire systems has further increased using rectangular nickel-titanium wires.[10] It is possible to change the form of preformed nickel-titanium wires with cold forming or by using a heat source. But these techniques are not recommended because of inducing significant changes in force level of the wire.[9] Some orthodontists do not care about the size of preformed nickel-titanium wires, since they believe the original arch size and arch shape will return back after using stainless steel arch wires with appropriate size and shape. This method is not recommended because it causes round tripping movement of the teeth during treatment and increases the subsequent side effects.[11]

Dental arches vary in different races and populations.[2, 4-5] Therefore arch wires should be selected according to the related population’s arch size and arch shape. In a study of American patients by Braun *et al.*, thirty three preformed nickel-titanium wires were compared with normal dental arches. They reported that the intercanine and intermolar widths of upper and lower preformed arch wires were larger than the average dental arch widths in almost their entire sample.[11] Similar results were achieved by another study conducted in India. The average intermolar width exceeded the average dental arch width by 2.893 mm in the maxillary arches and 1.861 mm in the mandibular arches. The average intermolar-intercanine width ratios for natural arches (2.11 for mandibular and 1.75 for maxillary) were greater than the ratios for the wire-bracket assemblies (1.78 for mandibular and 1.75 for maxillary).[5] According to a study conducted by Tulin Taner *et al. *in Turkey, maxillary and mandibular arch widths increased during orthodontic treatment. Arch form in both maxilla and mandible of Turkish samples in that study was tapered before the treatment. Maxillary arch forms changed in 81% of samples during the treatment due to using arch wires incompatible with the patients' arch form.[3] Contrary to the studies mentioned before, Souichiro Oda *et al.* revealed that preformed nickel-titanium wires were significantly narrower than Japanese dental arch in canine and molar regions. The preformed arch wires were approximately 1 to 3mm narrower at the canine level and 2 to 5 mm narrower at the first molar level.[7]

Because most of the available arch wires in Iran are designed according to normal dental arches of European and American population, this study was undertaken to compare the commercially available preformed nickel-titanium arch wires with the Iranian Angle class I normal occlusion dental arches and introduce the highest correlated arch wires with Iranian dental arch size and shape. 

## Materials and Method

Our study samples were 148 orthodontically untreated students, including 67 male and 81 female subjects. They were selected through convenient sampling among the students of Shiraz University of Medical Sciences. The inclusion criteria were having skeletal and dental class I, normal vertical growth pattern, normal overjet (1-2 mm) and overbite (2-3 mm), aligned teeth with minimum crowding and symmetric lower dental arch (checked with a transparent ruled grid). Normal skeletal class I pattern and normal vertical growth pattern were determined based on the clinical examination of the profile, and dental class I was selected based on class I canine and molar relationships. Subjects with the history of orthodontic treatment or posterior cross bite were excluded from the study. None of the selected cases had supernumerary or missing teeth or anterior teeth restorations. 

Samples were asked to sit upright and look at a distant point. For checking maxillary and mandibular prognathism or retrognathism, a true vertical line was imagined from glabella. Subnasale had to be 6±3 mm from this line and pogonion had to lie on or close to this line (0±4mm). Upper face was measured by glabella-subnasale distance, whereas lower face was measured by subnasale-menton. The upper face and lower face were equal in all the selected cases.

Study models of mandibular arch were provided and scanned using HP Scanner (Scanjet G4010; HP Company, China) with 600 dpi resolution. We used Cast Analyzer Iranian X Software (Khallaghane Mehr Co.; Tehran, Iran)[13] written in visual basic language for analyzing the casts. As it has been examined in a previous study of this software, the mean measurement error of the software was 0.1; thus, the measurement accuracy of the software was acceptable.

The twelve commercially-available preformed nickel-titanium arch wires in Iran were American orthodontics form 1, 2 and 3 (American Orthodontics; Sheboygan, WI), Masel (Masel Orthodontics; Carlsbad, CA), TruForce Ortho Technology (Ortho Technology; Tampa, FL), Ortho Organizer (Ortho Organizer; Carlsbad, CA), Astar (Astar; Shanghai, China), G&H Europa form 1 (G&H Orthodontics; Franklin, IN), Gestenco (Gestenco International AB; *Göteborg*, Sweden), ODP (ODP Inc.; Vista, CA), GAC (GAC International; Bohemia, NY), and Dentaurum (Dentaurum; Ispringen, Germany). Three arch form templates, ovoid, tapered and square (OrthoForm ^TM^; 3M, Unitek, CA, USA) were also scanned.

**Figure 1:**
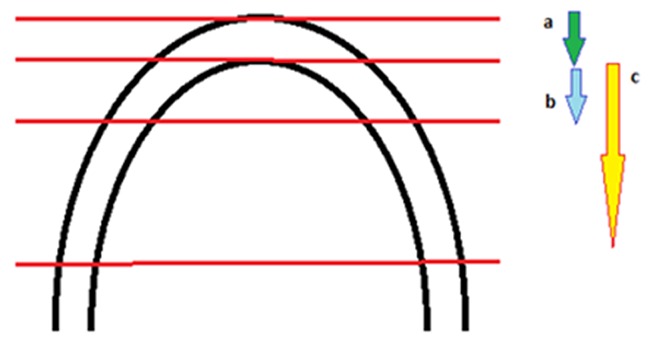
Calculation of arch wire depth.  a: thickness of incisor brackets, b: Average canine depth of population (measured from labial surface of lower incisors to midbuccal point of canines), c: Average molar depth of population (measured from labial surface of lower incisors to midbuccal point of first molars). Canine depth of arch wire equals average canine depth of population plus lower incisor bracket thickness. Inter-molar depth of arch wire equals average molar depth of population plus lower incisor bracket thickness.

Cast analysis was performed in order to select the most appropriate arch wire as well as to determine the arch shape for each subject. The indices, including intermolar width, intercanine width, canine depth, and molar depth were measured during these two stages to achieve higher accuracy (Figure 1).

In the first step of analysis, “arch wire curve analysis” option of the program was chosen. In the second step canine and first molars were selected as reference teeth, and mesial and distal points of each reference tooth were determined. In the next step, mid buccal points of each reference tooth and lower arch midline were determined. The mid buccal point is exactly the point on the buccal surface of the tooth over which the bracket base is placed. Brackets thicknesses were added to the buccal surface of the teeth according to averages given in a study by Souichiro Oda *et al.*[7] Means and standard deviations of brackets thicknesses at the mandibular central incisor, canine and first molar were 1.34±0.16, 0.75±0.11 and 0.73±0.08 mm, respectively. Then, dental arch measurements such as inter-canine width, inter-molar width, canine depth, and molar depth were calculated. Finally, dental arch size and form of each subject was determined and compared with those of twelve preformed nickel titanium arch wires. Cast Analyzer X Software selected the most appropriate arch wire for each cast by means of sixth degree polynomial function.

The Scanned subject’s dental arches were compared with three arch form templates of 3M Unitek (ovoid, tapered, square) in order to categorize dental arches into three groups. Averages arch widths and depths for each case were calculated. 

In the next part of the investigation, arch wire widths were compared with the average of intercanine and intermolar of the subjects. Intercanine and intermolar widths of arch wire were measured at its canine depth and molar depth. Canine depth of the arch wire equals average canine depth of population plus the lower incisor bracket thickness (1.34 mm). The molar depth of the arch wire equals average molar depth of population plus the lower incisor bracket thickness (1.34 mm). These measurements were performed using image analyzing software (Figure 1).

The statistical analyses were performed using SPSS software (version 16; SPSS Inc., Chicago, IL, USA). Means and standard deviations of intermolar width, intercanine width, canine depth, and molar depth were calculated. The normality assumption of variables was assessed using one–sample Kolmogorov-Smirnov test. Student’s t-test was used for comparing arch widths and arch depths of male and female subjects. Level of significance was set at 0.05. Coefficient of variation (SD/mean) was used to determine the variability of indices in our study sample. 

## Results

The most frequent arch form in the study population was tapered (45%). Square and ovoid arch forms were respectively the next common arch forms (Table 1). The most common arch wires for each arch shape are presented in Table 1. The same order of arch form frequency was seen in male and female groups.

Table 2 demonstrates the maximum, minimum, mean, standard deviation and coefficient of variation of intercanine widths, intermolar widths, canine depths and molar depths of dental arches in the study group. Among these four measurements, canine depth was the most variable index in the population, according to the coefficient of variation. Kolmogorov-Smirnov test indicated that all the four variables had normal distribution.

**Table 1:** Distribution of different arch forms in Iranian population and the most appropriate arch wire for each arch form

**Arch form**	**Number (percent)**	**Most common arch wire (percent)**
Form1(tapered)	67(45%)	Ortho Organizer (73%)
Form2(square)	48(32.5%)	ODP (27%)
Form3(ovoid)	33(22.5%)	American Orthodontics form2 (48%)
Total	148(100%)	

**Table 2:** Dental arch measurements in study dental casts of cases with normal occlusion

	**Minimum**	**Maximum**	**Mean**	**Standard Deviation**	**Coefficient of variation (SD/Mean)**
Intercanine width	20.1	36.1	27.803	2.0944	0.075
Intermolar width	41.9	57.7	49.489	3.2598	0.065
Canine depth	1.8	8.1	5.195	1.1197	0.21
Molar depth	20.7	33.6	26.697	2.2306	0.083

The mean of intermolar width in males (50.50±3.08) was statistically greater than females (48.64±3.19) (*p*< 0.001). There were no significant differences between the sex groups regarding the other three indices (Table 3).

**Table 3:** Dental arch dimensions in male and female subjects

	**Male** **(Mean± SD)**	**Female** **(Mean±SD)**	**P value**
Intercanine width	27.97±1.81	27.65±2.31	0.37
Intermolar width	50.50±3.08	48.64±3.19	<0.001
Canine depth	5.30±1.21	5.14±1.02	0.388
Molar depth	27.05±2.39	26.42±2.08	0.092

Comparing various dimensions of subjects' dental arch with those of twelve commercially selected arch wires revealed that the Ortho Organizer arch wire was the most appropriate available arch wire in 39% of cases. American Orthodontics form 2 and GAC arch wires matched 15% and 11% of cases, respectively. Correlation of commercially available arch wires with Iranian arch size and arch form is represented in Table 4. Ortho organizer arch wire was matched with 73% of cases who had tapered arch form.

**Table 4:** Number and percentage of different brands of arch wires matched with normal Iranian dental arches

**Arch wire**	**Number (percent)**
Ortho Organizer	58(39%)
American Orthodontics Form 2	22(15%)
Gac	16(11%)
ODP	15(10%)
American Orthodontics Form 1	10(7%)
Masel	6(4%)
Astar	5(3%)
G&H Europa	5(3%)
Gestenco	3(2%)
American Orthodontics Form 3	3(2%)
TruForce Orthotechnology	3(2%)
Dentaurum	2(1%)
Total	148

**Figure 2:**
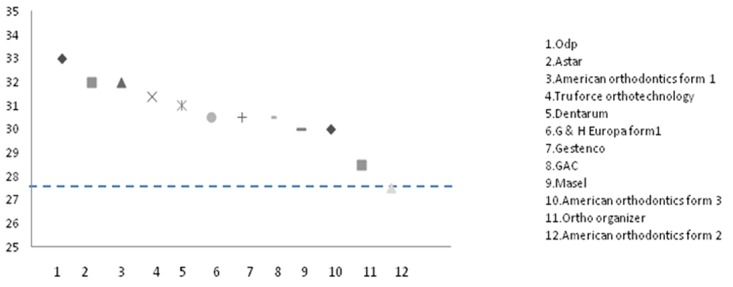
Mean of intercanine width of Iranian population in comparison with different arch wires. Dashed line refers to mean.

**Figure 3:**
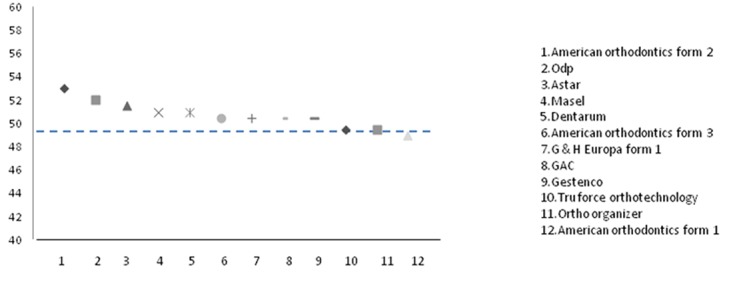
Mean of intermolar width of Iranian population in comparison with different arch wires. Dashed line refers to mean.

Figures 2 and Figures 3 have been designed to display the distribution of wires according to canine widths and molar widths in comparison with the mean of the dental arch (including mean brackets thicknesses). Most of the selected arch wires were wider at canine and molar level when compared with the average intermolar and intercanine width in normal study subjects. TruForce Ortho Technology and Ortho Organizer had the same intermolar widths as the average intermolar width of dental arch at the first molar level.

## Discussion

Ethnicity is an important factor that influences the shape and dimension of dental arches.[2, 4-5] Different ethnic populations have various dental arch dimensions. Our available preformed orthodontic arch wires are designed and fabricated in other countries based on the mean of arch size and shape of the manufacturers' own population. Therefore, it is necessary to compare dental arch dimensions of our population with those of commercially available arch wires for selecting more appropriate arch wires. Moreover, this will reduce the risk of post orthodontic occlusal relapse and lead into longer post treatment retention and more stability.

Studies conducted by Uysal *et al.*[14-15] in Turkey revealed that the mandibular arch widths of class II division I and II cases were significantly smaller than normal occlusion sample whereas class III cases had wider dental arches. Our samples were selected from class one occlusion students with normal vertical dimensions. Some previous studies have indicated that the relapse potential is related to orthodontic changes in dental arch dimensions, particularly in the lower intercanine area.[16-17] Therefore, maintaining the mandibular intercanine width during treatment leads to more stable orthodontic results. That was why the mandibular arch was selected for the current study.

Dental arch width changes during life; therefore, the arch width should be measured separately in each age group.[7] In this study subjects were selected from the adult group amongst whom changes in the arch size are negligible.

Some previous studies have only used the mean of Intercanine and intermolar width for selecting the most appropriate arch wires.[5, 7] Finding arch wires that match most dental arches would be a more valuable method than considering the mean for choosing the appropriate arch wires. We compared twelve different arch wires with each of 148 dental arches in order to find the best fitted arch wires.

There are different arch forms in every population. In a study by Bayome *et al.*[18] dividing dental arches into five arch forms instead of three arch forms produced no clinically significant differences. Hence, the classification based on three major arch forms seems more advantageous for clinical applications. The most frequent arch shape and average arch size of different ethnicities must be considered when selecting arch wires because each company manufactures arch wires according to the normal dental arch size and shape of a special population. Tapered arch form is the most common arch type among Turkish and Malaysians;[3-4] while for Korean people, the most frequent arch form is square.[2] Tapered arch form was the most common type in our population (45%) followed by square (32.5%) and ovoid shape (22.5%), respectively.

 The most appropriate arch wires for tapered, square and ovoid arches were also determined in the study. 

Ortho Organizer^TM^ was the most appropriate arch wire because this brand matched the tapered arch form, the most frequent mandibular arch shape in our normal population. For 39% of cases Ortho Organizer^TM^ was the best choice due to the proximity of intercanine and intermolar arch wire widths to the average intercanine and intermolar widths among the normal Iranian population. Most of the selected arch wires were wider at canine and molar levels when compared with the average intermolar and intercanine width in normal study subjects. A similar study of Indian dental arch dimensions revealed the same results,[5] whereas in other studies conducted in Japan the average width of preformed arch wires was found to be narrower than the mean of Japanese dental arches.[9, 12]

In spite of the availability of various brands of arch wires in Iran, only a few of them can be used safely to avoid post treatment instability. These facts suggest that manual arch wire adjustments may be necessary for prevention of side effects of stainless steel arch wires with inappropriate width. An orthodontist must be able to form suitable arch wires for each patient. Arch shape and arch widths in patients with class III, class II, long face and short face tendency are different from the normal population. Thus, further studies to compare preformed arch wires with these patients are required.[14-15] Also extraction cases with severe crowding may need special preformed nickel-titanium arch wires due to their smaller arches.

In the near future when digital models will eventually replace dental casts, arch wire selection with software may become one of the steps in designing treatment for each patient and using custom made arch wires may become frequent in patients with dentofacial deformity and cleft lip and palate patients. 

## Conclusion

Most of the preformed arch wires were wider in both intercanine and intermolar width than the average widths of our population dental arch. The variation in available preformed arch wires does not entirely cover the range of diversity of the normal dental arch. Narrow arch wires with tapered shape are better consistent with the Iranian lower arch. 
